# Lapatinib increases motility of triple-negative breast cancer cells by decreasing miRNA-7 and inducing Raf-1/MAPK-dependent interleukin-6

**DOI:** 10.18632/oncotarget.5700

**Published:** 2015-10-12

**Authors:** Yu-Chun Hsiao, Ming-Hsin Yeh, Yun-Ju Chen, Ju-Fang Liu, Chih-Hsin Tang, Wei-Chien Huang

**Affiliations:** ^1^ The Ph.D. program for Cancer Biology and Drug Discovery, China Medical University and Academia Sinica, Taichung, Taiwan; ^2^ Department of Surgery, Chung-Shan Medical University Hospital, Taichung, Taiwan; ^3^ Department of Medical Research, E-Da Hospital, Kaohsiung, Taiwan; ^4^ Department of Biological Science & Technology, I-Shou University, Kaohsiung, Taiwan; ^5^ Central Laboratory, Shin-Kong Wu Ho-Su Memorial Hospital, Taipei, Taiwan; ^6^ Graduate Institute of Basic Medical Science, China Medical University, Taichung, Taiwan; ^7^ Department of Pharmacology, School of Medicine, China Medical University, Taichung, Taiwan; ^8^ Department of Biotechnology, College of Health Science, Asia University, Taichung, Taiwan; ^9^ Center for Molecular Medicine, China Medical University and Hospital, Taichung, Taiwan; ^10^ Graduate Institute of Cancer Biology, China Medical University, Taichung, Taiwan

**Keywords:** lapatinib, IL-6, migration, microRNA, Raf-1

## Abstract

Lapatinib, a dual epidermal growth factor receptor (EGFR) and HER2 tyrosine kinase inhibitor (TKI), has been approved for HER2-positive breast cancer patients. Nevertheless, its inhibitory effect on EGFR did not deliver clinical benefits for triple-negative breast cancer (TNBC) patients even EGFR overexpression was frequently found in this disease. Moreover, lapatinib was unexpectedly found to enhance metastasis of TNBC cells, but the underlying mechanisms are not fully understood. In this study, we explored that the level of interleukin-6 (IL-6) was elevated in lapatinib-treated TNBC cells. Treatment with IL-6 antibody abolished the lapatinib-induced migration. Mechanistically, the signaling axis of Raf-1/mitogen-activated protein kinases (MAPKs), c-Jun N-terminal kinases (JNKs), p38 MAPK, and activator protein 1 (AP-1) was activated in response to lapatinib treatment to induce IL-6 expression. Furthermore, our data showed that microRNA-7 directly binds and inhibits Raf-1 3′UTR activity, and that down-regulation of miR-7 by lapatinib contributes to the activation of Raf-1 signaling pathway and the induction of IL-6 expression. Our results not only revealed IL-6 as a key regulator of lapatinib-induced metastasis, but also explored the requirement of miR7/Raf-1/MAPK/AP-1 axis in lapatinib-induced IL-6 expression.

## INTRODUCTION

Breast cancer is one of the leading cancers in women and the incidence gradually increases year by year [1]. According to the expression status of hormone and HER2 receptors, breast cancers have been further classified into luminal and HER2-positive subtypes, respectively. In contrast to these subtypes, the “basal-like” subtype of tumors is composed almost entirely of the triple-negative breast cancer (TNBC), which is negative for the expressions of estrogen and progesterone receptors (ER and PR) and HER2 [2, 3]. Population-based studies have demonstrated that TNBC patients showed lower survival rate as compared to those with luminal phenotypes [2, 4, 5]. TNBC patients were much more likely to develop a recurrence during the first 3 years after treatment and showed a consistent rate of recurrence over the follow-up period [2, 5]. Recent studies suggest that TNBC patients have a higher visceral metastasis, such as brain metastasis [2].

Scientific efforts aiming at dissecting the cancer biology of TNBC have revealed several promising therapeutic targets including epidermal growth factor receptor (EGFR), a critical membrane-bound receptor tyrosine kinase for cancer progression. EGFR expression has been detected in up to 80% of TNBC [6, 7]. Lapatinib is an oral dual tyrosine kinase inhibitor (TKI) inhibiting both EGFR and HER2 receptors. Although the majority of clinical benefits from lapatinib were achieved in patients with HER2-positive breast cancers, treatment with lapatinib for TNBC or HER2-negative patients continues to be of interest due to its anti-EGFR activity [8, 9]. Thus, lapatinib has also been tested as monotherapy or in combination with other systemic therapies for triple-negative or other HER2-negative breast cancers in phase II trials [10, 11]. Unfortunately, lapatinib has shown a lack of dramatic efficacy in overall HER2-negative diseases, suggesting that EGFR tyrosine kinase activity may not be the major vulnerability in TNBC. Surprisingly, treatment with lapatinib in triple-negative or HER2-negative/PR-negative breast cancer patients even showed a worse clinical outcome with shorter median event-free survival [8, 12–14]. Our previous studies further uncovered an off-target activity of lapatinib in promoting the aggressiveness of TNBC cell lines through induction of EGFR and cyclooxygenase-2 (COX-2) expressions [15, 16]. The increase in EGFR expression is attributed to the down-regulation of microRNA-7, and histone deacetylase (HDAC) inhibitor trichostatin A (TSA) could suppress this event through restoring of the microRNA-7 level [17]. The increased EGFR protein in turn cooperates with HuR to enhance COX-2 expression through mRNA stabilization. In addition to EGFR, elevation of NF-κB activity through Src-dependent pathway has also been demonstrated to contribute to the lapatinib-increased COX-2 expression [16]. However, other mechanisms, in addition to COX-2 up-regulation, responsible for the lapatinib-induced aggressiveness of TNBC cannot be excluded.

Accumulating evidences have revealed that the inflammation-related cytokines contribute to the processes of tumor formation and progression and may counteract to the efficacy of cancer therapy [18–21]. During the tumorigenesis, pro-inflammatory cytokines are frequently up-regulated by oncogene-driven signaling pathways in the tumor microenvironment and further alter the epithelial-mesenchymal transition (EMT) property of tumor cells. Under this condition, tumor cells become more aggressive and resistant to cancer therapy [22–28]. Interleukin-6 (IL-6) has been reported to play a major role in these processes [21]. Our previous study have shown that lapatinib treatment increases the IL-6 transcription in TNBC cells [16]. However, it remains unknown whether and how IL-6 expression contributes to the lapatinib-induced aggressiveness of TNBC cells.

In this study, our results showed that IL-6 production was dramatically induced and contributed to the increase in migration ability of MDA-MB-231 TNBC cells in response to lapatinib treatment. Mechanistically, lapatinib induces IL-6 expression through Raf-1/MAPK/AP-1 signaling axis by down regulating microRNA-7. These findings not only provide a new insight of lapatinib-induced motility of TNBC cells, but also suggest that targeting IL-6 may be a potential strategy to reduce the recurrence and metastasis of breast cancer in combination with EGFR/HER2 TKIs.

## RESULTS

### IL-6 expression was up-regulated in lapatinib-treated TNBC cells

**Figure 1:**
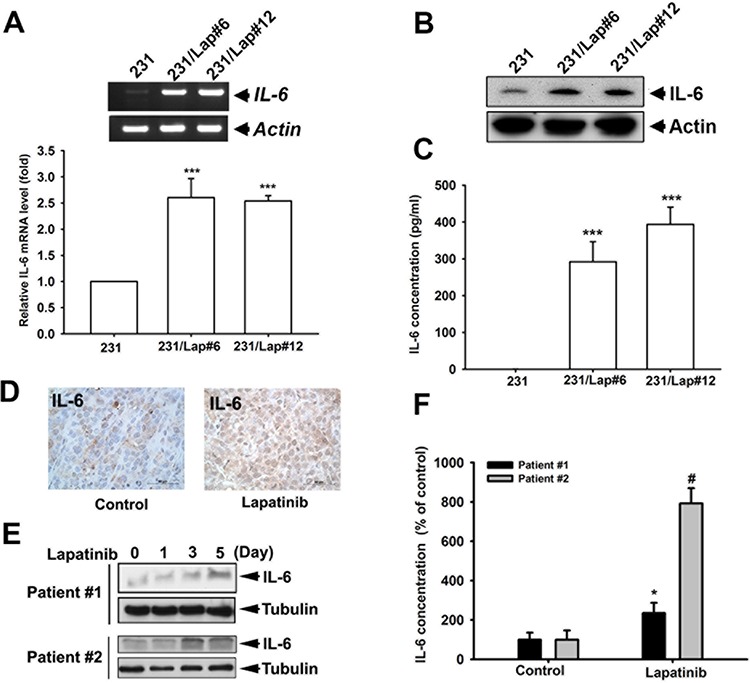
IL-6 expression was up-regulated by lapatinib in MDA-MB-231 TNBC cells **A.** Total RNA extracted from MDA-MB-231 (231) cells and their lapatinib-treated clones (231/Lap) was applied to examine the IL-6 mRNA level in both general RT-PCR (*upper panel*) and quantitative RT-PCR (*lower panel*) analysis. **B.** The whole cell lysates of 231 and their lapatinib-treated clones were harvested and subjected to western blot analysis to examine the protein expression of IL-6. **C.** The incubated media from 231 cells and 231/Lap cells were collected and subjected to ELISA for detection of IL-6 protein secretion. **D.** IL-6 expression in tumor sections of MDA-MB-231 xenograft mouse receiving lapatinib treatment was determined in immunohistochemical staining with specific anti-IL-6 antibody. **E.** The whole cell lysates of primary human breast cancer cells treated with or without lapatinib for indicated time were harvested and subjected to western blot analysis to examine IL-6 protein expression. **F.** The incubated media from primary human breast cancer cells from (E) were collected and subjected to enzyme-linked immunosorbent assay (ELISA) for detection of IL-6 protein secretion. Results were expressed as mean ± S.E.M of three independent experiments. **: *p* < 0.01; ***: *p* < 0.001; #: *p* < 0.05 as compared with control group.

Our previous study showed that TNBC cells become more aggressive in response to long-term treatment with lapatinib [15]. To investigate whether IL-6 is critical for the lapatinib-induced aggressiveness of TNBC cells, we first examined the IL-6 expression in both TNBC MDA-MB-231 (231) cells and MDA-MB-231/Lap (231/Lap) clones, which were established by long-term treatment of 231 cells with lapatinib [16]. The mRNA expression of IL-6 was obviously elevated in 231/Lap#6 and 231/Lap#12 clones as compared with the parental 231 cells (Figure 1A). In parallel, IL-6 protein level detected by Western blot analysis was increased in these 231/Lap cells (Figure 1B). The secretion of IL-6 into the medium from 231/Lap clones, detected in enzyme-linked immunosorbent assay (ELISA), was also higher than that from parental 231 cells (Figure 1C). In support to this finding *in vivo*, IL-6 expression was higher in lapatinib-treated MDA-MB-231 xenograft tumors in SCID mice (Figure 1D), which showed aggressive metastasis to axillary lymph nodes in our previous study [15]. The inductions of total or secreted IL-6 level by lapatinib in a time-dependent manner were further found in primary human breast cancer cells from two TNBC patients (Figure 1E and 1F, respectively). These results indicate that lapatinib treatment induced IL-6 expression in TNBC cells.

### Elevation of IL-6 contributes to the lapatinib-enhanced aggressiveness of TNBC cells

**Figure 2:**
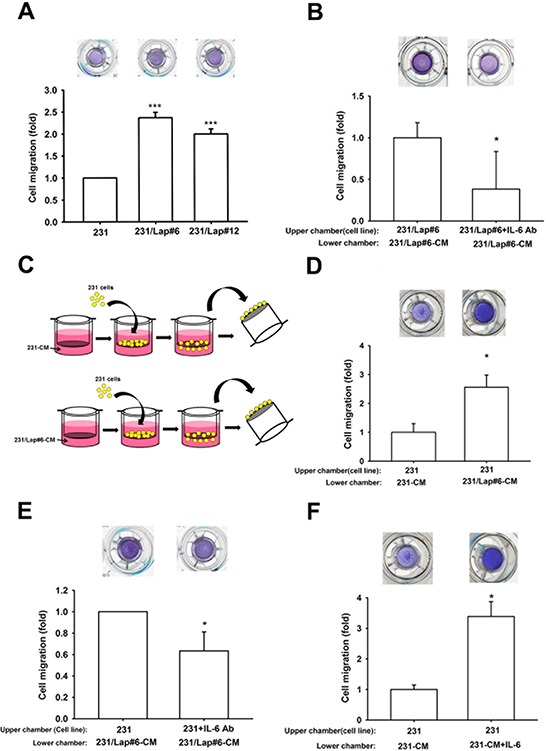
IL-6 is involved in the lapatinib-enhanced aggressiveness of 231 cell **A.** In transwell migration assay, the 231 and 231/Lap cells were seeded in upper chamber with serum-free medium. The migrated cells through the membrane were stained with crystal violet and counted. **B.** The 231/Lap#6 cells were treated with or without anti-IL-6 antibody (30 ng/ml) in the upper chamber for 24 hrs, and then migrated cells were counted in transwell migration assay. **C.** and **D.** As illustrated in (C), the incubated media from 231 cells and 231/Lap#6 cells were collected and defined as conditioned media 231-CM and 231/Lap#6-CM, respectively. These conditioned media were added into the lower chamber. The 231 cells were seeded in the upper chamber with serum-free medium. The migrated 231 cells was further analyzed and quantified. **E.** The 231/Lap#6 cells were treated with or without neutralizing IL-6 antibody (30 ng/ml). The lower chamber was filled with 231/Lap#6-CM. The migrated 231/Lap#6 cells were further analyzed and quantified. **F.** The 231 cells were treated with or without recombinant IL-6 protein (30 ng/ml) in the lower chamber of transwell migration assay. The quantitative results were expressed as mean ± S.E.M of three independent experiments. *: *p* < 0.05; ***: *p* < 0.001 as compared with control group.

Up-regulation of IL-6 has been reported to enhance the migration ability of various tumor cells [21, 26, 32]. In consistence with our previous findings [15], lapatinib-treated clones (231/Lap#6 and 231/Lap#12) showed higher migration ability than their parental cells in transwell migration assays (Figure 2A). We next examined whether IL-6 secretion is involved in lapatinib-induced migration ability of TNBCs through an autocrine regulation. To this end, 231/Lap#6 cells were incubated with or without specific anti-IL-6 antibody in the transwells. The results showed that their migration ability was dramatically inhibited by anti-IL-6 antibody (Figure 2B). To further confirm this finding, 231 and 231/Lap#6 cells were cultured for 24 hrs, and then the incubated media were collected and defined as conditioned media 231-CM and 231/Lap#6-CM, respectively. As illustrated in Figure 2C, these conditioned media were added into the lower chamber of transwells, and 231 cells in serum-free medium were seeded in the upper chamber for migration assay. Incubation with 231/Lap#6-CM induced higher migration ability of 231 cells than that with 231-CM did (Figure 2D) and this effect was attenuated by anti-IL6 antibody (Figure 2E). Furthermore, the migration ability of 231 cells was also enhanced when recombinant IL-6 protein was added into 231-CM (Figure 2F). These results indicated that lapatinib enhances the migration ability of TNBC cells through IL-6 up-regulation.

### MAPK/AP-1 axis mediated lapatinib-induced IL-6 production in TNBC cells

**Figure 3:**
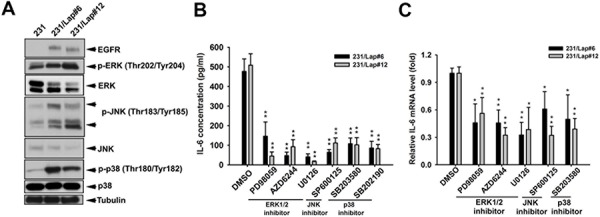
Lapatinib induced IL-6 expression through MAPK signaling pathways **A.** Total proteins extracted from 231 and 231/Lap cells were subjected to western blotting analysis with indicated antibodies. **B.** and **C.** 231/Lap cells were treated with PD98059 (10 μM), U0126 (10 μM), AZD6244 (500 nM), SP600125 (30 μM), SB203580 (20 μM) and SB202190 (10 μM) for 2 days, followed by examination of IL-6 protein (B) and mRNA (C) expressions in ELISA and RT-qPCR analyses, respectively *: *p* < 0.05; **: *p* < 0.01 as compared with control group.

We next investigated how the IL-6 expression is up-regulated by lapatinib. Accumulating evidences showed that IL-6 expression is mainly controlled by MAPK pathway [33–35], and sustained activation of MAPK pathways was observed in response to lapatinib resistance in HER2-positive breast cancer cells [36]. We thus examined the involvement of MAPK pathways in the up-regulation of IL-6 expression in 231/Lap cells. As shown in Figure 3A, activities of ERK1/2, p38, and JNK were higher in 231/Lap cells compare to 231 cells. Pharmacological inhibitors of individual MAPKs (PD98059, AZD6244 and U0126 for ERK1/2; SP600125 for JNK; SB203580 and SB202190 for p38) suppressed the IL-6 expression at both protein (Figure 3B) and mRNA levels (Figure 3C), suggesting that lapatinib induces IL-6 expression in part through activation of MAPK pathways.

**Figure 4:**
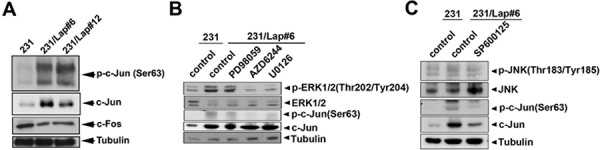
c-Jun activation is involved in lapatinib-induced IL-6 production **A.** Total proteins extracted from 231 and 231/Lap cells were subjected to western blotting analysis with specific antibodies against c-Fos and c-Jun. **B.** and **C.** 231 and 231/Lap#6 cells were pre-treated with or without ERK pathway inhibitors PD98059, U0126, AZD6244 (B), and JNK pathway inhibitor SP600125 (C) for 2 days, followed by total protein extraction and western blotting analysis with indicated antibodies.

**Figure 5:**
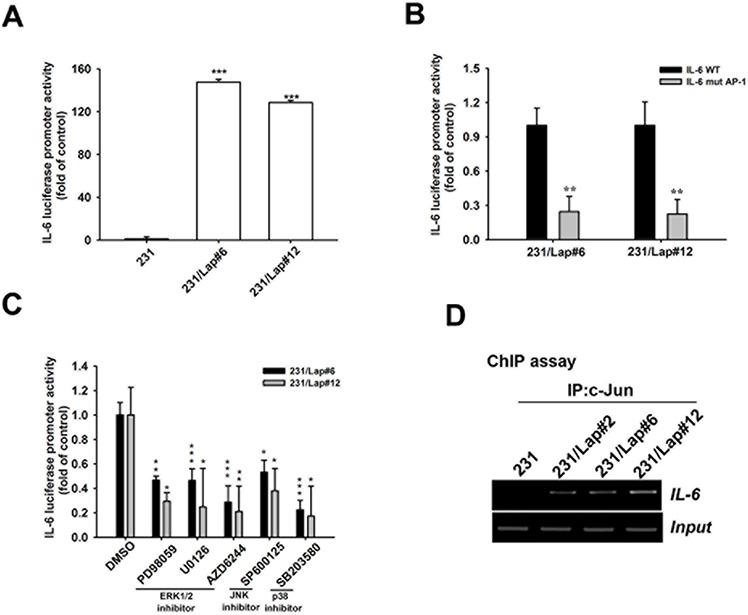
MAPK/AP-1 axis mediates lapatinib-induced IL-6 gene transcription **A–C.** 231 or 231/Lap cells were transfected with wt (A) and/or AP-1 mutated (B) IL-6 promoter luciferase reporter gene for 2 days followed by treatment with MAPK pathway inhibitors (C), and then total lysates were prepared and subjected to luciferase activity assays. **D.** Total lysates from 231 and 231/Lap cells were prepared and subjected to chromatin immunoprecipitation assay with anti-c-Jun antibody to detect the DNA binding activity of c-Jun on *IL-6* promoter. One percent of the precipitated chromatin was used to verify the equal sample loading (input). *: *p* < 0.05; **: *p* < 0.01; ***: *p* < 0.001 as compared with control group.

Transcription factor AP-1 is consist of c-Jun and c-Fos and is controlled by MAPK pathways [37–39]. Since MAPK pathways mediate the IL-6 expression in 231/Lap cells, we further addressed whether the *cis*-regulatory element of AP-1 on *IL-6* promoter region is required for lapatinib-induced IL-6 expression. The protein expression and activation of c-Jun, but not c-fos, by lapatinib were found in 231/Lap#6 cells (Figure 4A), and ERK1/2 inhibitors PD98059, U0126 and AZD6244 significantly inhibited the phosphorylation of c-Jun (Figure 4B). Suppression of JNK by SP600125 also dramatically reduced both the protein and phosphorylation level of c-Jun in 231/Lap#6 cells (Figure 4C). These data suggest that lapatinib-activated MAPK pathways regulate IL-6 expression via AP-1 activation. Indeed, IL-6 promoter activity was higher in 231/Lap cells than in parental 231 cells (Figure 5A). Mutation of AP-1-binding sites (Figure 5B) and MAPK inhibitors (Figure 5C) significantly decreased the promoter activity. Furthermore, the binding activity of c-Jun protein on the promoter region of *IL-6* gene as detected by chromatin immunoprecipitation assay was dramatically increased in 231//Lap cells (Figure 5D). These results demonstrated that treatment with lapatinib induces MAPK pathways and c-Jun expression to turn on *IL-6* gene transcription.

### MicroRNA-7 downregulation contributes to lapatinib-induced Raf-1 activation in mediating MAPK/AP-1 activation and IL-6 production

**Figure 6:**
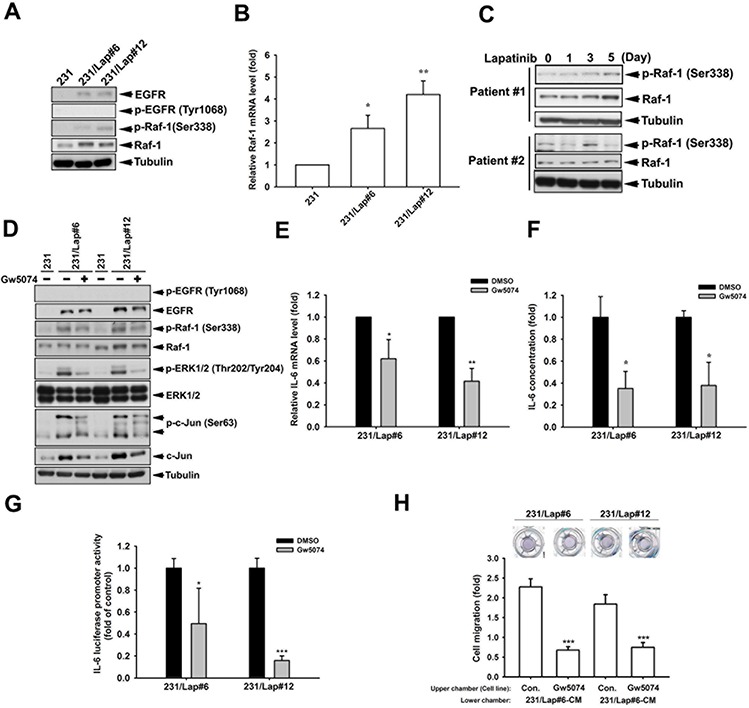
Raf-1 activation mediates *IL-6* gene expression in 231/Lap cells **A.** Total proteins extracted from 231 and 231/Lap cells were subjected to western blotting analysis with specific antibodies against Raf-1 and p-Raf-1. **B.** Total RNA extracted from 231 and 231/Lap cells was applied to examine the Raf-1 mRNA level in quantitative RT-PCR analysis. **C.** Total proteins extracted from 231 cells treated with lapatinib for indicated days were subjected to western blotting analysis with specific antibodies against Raf-1 pathway. **D.** Total proteins extracted from 231 and 231/Lap cells treated with Raf-1 inhibitor were subjected to western blotting analysis with specific antibodies against Raf-1 pathway. **E.** Total RNA extracted from Gw5074-treated 231/Lap cells was applied to examine the IL-6 mRNA level in quantitative RT-PCR analysis. **F.** The incubated media from 231/Lap cells treated with Gw5074 was collected and subjected to ELISA for detection of IL-6 protein secretion. **G.** 231/Lap cells were transfected with wt *IL-6* promoter luciferase reporter gene for 2 days followed by treatment with Gw5074, and then total lysates were prepared and subjected to luciferase activity assays. **H.** The 231/Lap cells were treated with or without Gw5074 in the upper chamber for 24 hrs, and then migrated cells were counted in transwell migration assay. *: *p* < 0.05; **: *p* < 0.01; ***: *p* < 0.001 as compared with control group.

We next addressed the underlying mechanisms of MAPK/AP-1 activation in response to lapatinib treatment. Raf-1 activates the MAPK/ERK kinase (MEK) 1/2 dual-specificity protein kinases, which then activate MAPKs. Elevations of Raf-1 activity and protein expression (Figure 6A) as well as mRNA (Figure 6B) were found in 231/Lap clones. The induction of Raf-1 by lapatinib in a time-dependent manner was also found in human primary breast cancer cells from TNBC patients (Figure 6C). Raf-1 inhibitor GW5074 attenuated lapatinib-induced ERK1/2 and c-Jun phosphorylations in 231/Lap cells (Figure 6D), indicating the contribution of Raf-1 to the activation of MAPK/AP-1 axis. Furthermore, Gw5074 also suppressed the IL-6 mRNA (Figure 6E) and protein (Figure 6F) expressions in 231/Lap cells, and significantly decreased the promoter activity of *IL-6* gene (Figure 6G). More importantly, this inhibitor suppressed the migration ability of 231/Lap cells (Figure 6H). These results indicate the essential role of Raf-1 in lapatinib-induced IL-6 expression and migration.

**Figure 7:**
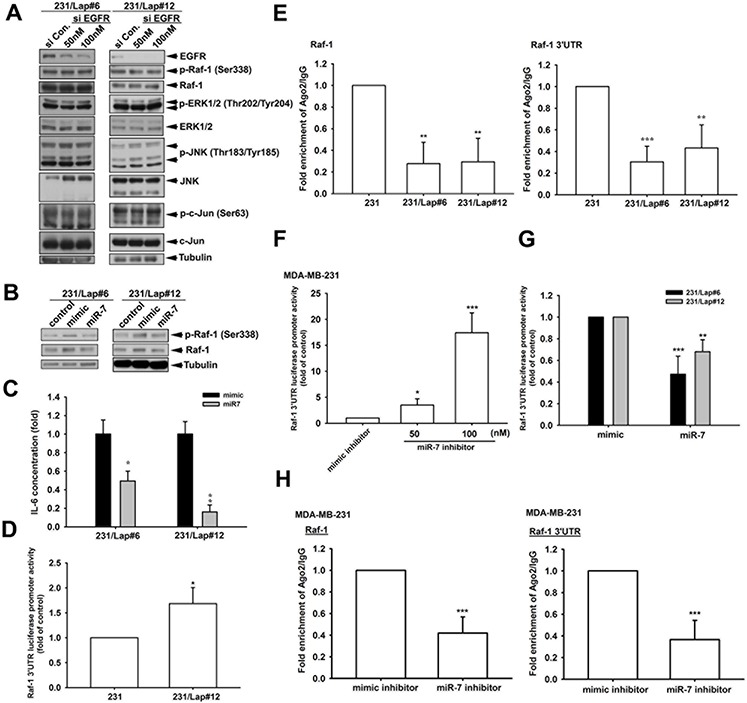
Elevation of Raf-1 expression in response to lapatinib is due to the de-repression from miR-7 **A.** and **B.** Total protein extracted from 231/Lap cells transfected with EGFR siRNA (A) or miR-7 (B) was subjected to Western blot analysis with indicated antibodies. **C.** The incubated media from 231/Lap cells transfected with miR-7 were collected and subjected to ELISA for detection of IL-6 protein secretion. **D, F, G.** 231 and 231/Lap cells were transfected with Raf-1 3′UTR luciferase reporter gene in the presence or absence of miR-7 mimic or inhibitor for 2 days, and then total lysates were prepared and subjected to luciferase activity assays. **E.** and **H.** Total lysates from 231 and 231/Lap cells transfected with or without miR-7 mimic or inhibitor were prepared and subjected to RNA immunoprecipitation assay with anti-ago2 antibody to detect the RNA binding activity of ago2 on Raf-1 mRNA with two specific primer sets. *: *p* < 0.05; **: *p* < 0.01; ***: *p* < 0.001 as compared with control group.

We next investigated the mechanism of Raf-1 upregulation in 231/Lap cells. Our pervious study showed an increase in EGFR expression in response to lapatinib treatment [16], raising the possibility that the upregulated EGFR induced the Raf-1 activation. However, knockdown of EGFR by siRNA did not affect the Raf-1 and ERK activations (Figure 7A), ruling out the involvement of EGFR. MicroRNAs (miRNAs) are involved in processes of cancer that includes development, differentiation, proliferation, and apoptosis [40–42]. Our previous study showed that lapatinib treatment reduced the expression of microRNA-7 (MiR-7) in MDA-MB-231/Lap cells, and thereby led to their enhanced migration and invasion abilities [16]. MiR-7 also functions in cell cycle arrest and in reducing cell growth and viability [43–45]. We thus investigated the role of miR-7 in mediating lapatinib-induced Raf-1/MAPK/c-Jun activation and IL-6 expression by restoring the expression of miR-7 in MDA-MB-231/Lap cells. As shown in Figure 7B, overexpression of miR-7 suppressed lapatinib-induced Raf-1 expression and phosphorylation. Restoration of miR-7 also reduced the IL-6 expression in MDA-MB-23/Lap cells (Figure 7C). Furthermore, Raf-1 3′ UTR activity was higher in 231/Lap cells than in parental cells as measured by luciferase-reporter assay (Figure 7D). Association with Ago2 is required for microRNA-mediated translation suppression. Binding of Ago2 on Raf-1 mRNA 3′UTR was lower in 231/Lap cells than in parental 231 cells (Figure 7E). To further confirm the inhibitory effect of miR-7 on Raf-1 3′UTR activity, miR7 inhibitor was transfected into MDA-MB-231 cells in the luciferase reporter assays, and results showed a dose-dependent increase in Raf-1 3′UTR luciferase activity (Figure 7F). In contrast, overexpression of miR7 mimic reduced Raf-1 3′UTR luciferase activity in 231/Lap clones (Figure 7G). In addition, our data reveled that the ago2 binding activity on Raf-1 3′UTR was also reduced by miR-7 inhibitor in RNA-IP analysis (Figure 7H). These results indicate that lapatinib treatment induced IL-6 production through de-repression of Raf-1 via downregulating miR-7.

## DISCUSSION

Triple negative breast cancers (TNBCs) express neither hormone receptors nor HER2 and possess highly invasive ability. Although chemotherapy is the major therapeutic option, recent scientific efforts have revealed that targeting EGFR may be a potential strategy for such patients. TNBCs frequently overexpress EGFR and the antitumor activity of lapatinib in such diseases was also tested [46, 47]. However, this drug did not show clinical benefits, and, in contrast, even promoted cancer metastasis and development of drug resistance [16].

Induction of reactive responses by cancer therapy has been proposed to increase oncogenic potential of cancer and lead to drug resistance [48–50]. The compensatory hypoxic responses in response to angiogenesis inhibition, including HIF-1-induced gene expression and secretion of angiogenic and autocrine growth factors, glycolitic enzymes, and extracellular proteases, may also promote metastasis and invasion [51, 52]. In metastatic tumor microenvironment, high level of pro-inflammatory cytokines in metastatic tumor microenvironment has been detected, and renders cancer cells highly transformative [20, 26]. IL-6 has been proposed to enhance cell proliferation and metastatic ability of tumor cells [20, 21, 26, 53]. Co-targeting of IL-6 and VEGF was found to potently inhibit glioma growth and invasiveness [54], implying that IL-6 production in response to cancer therapy may contribute to the invasiveness of recurrent tumors. Several lines of evidence in this study demonstrated that lapatinib treatment enhanced cell migration through induction of IL-6 expression. First, IL-6 protein and mRNA expressions are higher in lapatinib-resistant cells. Second, addition of IL-6 significantly induced cell motility. Third, neutralization of IL-6 by specific antibody in the culture medium reduced the migration of MDA-MB-231/Lap cells. These results suggest that the autocrined IL-6 up-regulated by lapatinib mediated the cell migration, and that targeting IL-6 may reduce the off-target effect of lapatinib on cell motility.

We previously reported that lapatinib induced migration and invasion of TNBC cells through COX-2 and EGFR expression via downregulation of microRNA-7 [26]. In this study, our data also demonstrate the involvement of miR-7 downregulation in lapatinib-increased Raf-1 activation and IL-6 expression, revealing multiple roles of miR-7 in response to lapatinib in TNBCs. Raf-1, belonging to the MKKK family, is regulated by multiple mechanisms including phosphorylation and interactions with various proteins, and functions as an upstream molecule of mitogen-activated protein kinase ERK, JNK and p38 pathways, which are involved in the regulation of gene expression [55]. IL-6 promoter activity was suppressed by the inhibitors of Raf-1, MEK, JNK and p38 in 231/Lap cells, indicating that IL-6 production is mediated by Raf-1/ MAPK activation in response to lapatinib treatment. DNA binding sites for AP-1 transcription factor, were found in the 5′ region of IL-6 gene [37–39]. Raf-1, MEK and JNK inhibitor also reduced the binding activity of c-Jun to the AP-1 element on IL-6 promoter and the promoter activity, showing that AP-1 activation contributes to lapatinib-induced IL-6 production in TNBCs. A recent study reported that specific EGFR tyrosine kinase inhibitor erlotinib induce IL-6 expression in head and neck squamous cell carcinoma through NOX4/p38 and JNK/ NF-κB pathway [28, 56]. Therefore, Raf-1/MAPK signaling pathway might be a common signaling pathway in IL-6 upregulation in response to EGFR tyrosine kinase inhibitors. However, it remains to be clarified whether these effects are due to inhibition of EGFR kinase activity.

**Figure 8:**
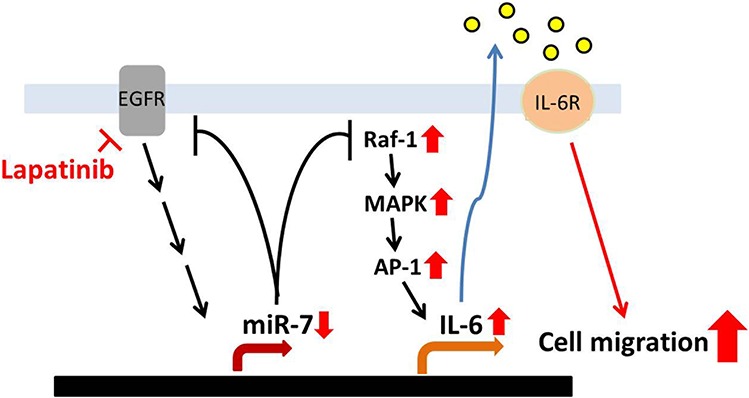
Illustration of lapatinib-enhanced aggressiveness via Raf-1/MAPK-dependent IL-6 expression Long-term treatment with lapatinib activates Raf-1/MAPK signaling pathways though downregulation of miR-7 expression. Then, the activated MAPKs induce expression and binding of c-Jun to *IL-6* promoter, which leads to the production of IL-6. The secreted IL-6 in turn enhances the migration ability of TNBC cells.

MicroRNAs (miRNAs) are involved in processes of carcinogenic including development, differentiation, proliferation, and apoptosis [40–42]. Aberrant expression and function of microRNAs have been associated with tumorigenesis. MiR-7 has been reported to down-regulate EGFR mRNA and other genes in lung breast, and glioblastoma cell cancer cell lines to induce cell cycle arrest and cell death [45, 57]. Our previous study reported that lapatinib enhances COX-2 and EGFR expression through downregulation of miR-7 [16]. In addition to being targeted by miR-7, EGFR has also been reported to induce miR-7 transcription relying on its tyrosine kinase activity [58], suggesting that miR-7 regulates EGFR expression through negative feedback. Therefore, inhibition of EGFR kinase activity by lapatinib may account for the downregulation of miR-7 and subsequent elevation of the Raf-1/ERK and IL-6 expression. In addition to its potential role in tumor promotion and progression, overexpression of IL-6 has been suggested to promote resistance to EGFR TKIs in both EGFR-dependent and EGFR-independent manners [59]. However, silencing EGFR expression did not affect Raf-1/MAPK/AP-1 protein levels in 231/Lap clones. Our data suggest that lapatinib induced Raf-1 expression due to downregulation of miR-7, which can bind to and target Raf-1 3′ UTR, leading to the downregulation of Raf-1 protein level. Transfection of lapatinib-treated cells with miR-7 also reduced lapatinib-increased IL-6 production and down-regulate Raf-1 activity. These results indicate that downregulation of miR-7 may contribute to activation of Raf-1/MAPK signaling pathway in breast cancer cells in response to lapatinib treatment (Figure 8).

In summary, our results elucidate a role of miR-7-dependent activation of Raf-1/MAPK/AP-1 in contribution to IL-6 upregulation and subsequent cell migration, suggesting a possible link to drug resistance. These findings imply IL-6 as a potential target for enhancing the therapeutic efficacy of lapatinib.

## MATERIALS AND METHODS

### Materials

Mouse polyclonal antibodies specific for IL-6, rabbit polyclonal antibodies specific for EGFR, phospho-c-Jun and c-Jun were purchased from Santa Cruz Biotechnology (Santa Cruz, CA). Rabbit polyclonal antibodies specific for phospho-Raf-1 Ser338, Raf-1, phospho-ERK1/2 Thr202/Tyr204, ERK1/2, phospho-JNK Thr183/Tyr185, JNK, phospho-p38 Thr180/Tyr182 and p38 were from Cell Signaling (Danvers, MA). PD98059, SB203580, SP600125, U0126, GW5074 and AZD6244 were obtained from Calbiochem (San Diago, CA). Mouse polyclonal antibody specific for IL-6 was purchased from R&D systems (Minneapolis, MN). All chemicals were obtained from Sigma-Aldrich (St Louis, MO). IL-6 enzyme immunoassay kit was purchased from Cayman Chemical (Ann Arbor, MI).

### Cell culture

Human TNBC MDA-MB-231 cell line and its lapatinib-treated derivatives were cultured in Dulbecco's modified Eagle's medium (DMEM)/F12 supplemented with 10% FBS, 100 unit/ ml penicillin and 100 mg/ml streptomycin. All cancer cell lines were maintained in a humidified 5% CO_2_ incubator at 37°C. The lapatinib-treated derivatives were established and selected by treating MDA-MB-231 cells with gradually increasing concentrations of lapatinib from 1 μM.

### Preparation of conditioned medium (CM)

MDA-MB-231 and its lapatinib-treated derivatives, MDA-MB-231/Lap, were grown to reach confluence. The culture media were refreshed with DMEM/F12. Conditioned media were collected 1 day after the refreshment of media and stored at −80°C until use as described previously [29, 30]. The MDA-MB-231-CM was used as reference.

### Measurement of IL-6 expression

MDA-MB-231 and MDA-MB-231/Lap cell lines were seeded in 6-well plates and allowed to grow for 24 hrs. Cells were pre-treated with various inhibitors for 48 hrs. Then, the medium was collected and stored at −80°C until use. IL-6 expression in the medium was assayed by using IL-6 enzyme immunoassay kits, according to the procedure described by the manufacturer.

### Reverse transcription-polymerase chain reaction (RT-PCR)

Total RNA was extracted from MDA-MB-231 and MDA-MB-231/Lap cells by using TRIzol reagent. The reverse transcription reaction was performed by using 1 ug of total RNA that was reversely transcribed into cDNA with oligo (dT) primer. The cDNA level of IL-6 was examined in quantitative PCR analysis with IL-6 specific primer, which sequences are as follows: Forward: 5′-TTGAGACTCATGGGAAAATCC-3′ and Reverse: 5′-CAAGACATGCCAAAGTGCTG-3′.

### Western blot analysis

Whole cell lysates were prepared as indicated experiments. Proteins were resolved on sodium dodecyl sulfate (SDS)-polyacrylamide gel electrophoresis and transferred to polyvinyldifluoride (Immobilon-P) membranes. The blots were blocked with 5% milk for 1 hr at room temperature and probed with rabbit antibodies against Raf-1, ERK1/2, JNK or p38 (1:1000) for overnight at 4°C. After washing three times, the blots were subsequently incubated with an anti-rabbit peroxidase-conjugated secondary antibody (1:10000) for 1 hr at room temperature. The blots were visualized by enhanced chemiluminescence with use of Kodak X-OMAT LS film.

### Transfection and reporter gene assay

TNBC cells were transfected with 1 μg of luciferase-bearing plasmid and 0.1 μg of β-galactosidase expression vector. TNBC cells were grown to 80% confluence in 12 well plates and subjected to transfection with *Trans*IT®-2020 on the following day. DNA and *Trans*IT®-2020 transfection reagent were pre-mixed with a ratio of 1:1 in serum-free medium for 20 mins and then applied to the cells. Serum was supplemented to the cells 6 hrs later. After 24- hr transfection, the cells were subjected to indicate experiments. Whole cell lysates were harvested with reporter lysis buffer (Promega) and the supernatants were collected after centrifugation at 13,000 rpm for 10 mins. Aliquots of cell lysates (20 μl) containing equal amounts of protein (20–30 μg) were placed into wells of an opaque black 96-well microplate. An equal volume of luciferase substrate was added to all samples and luminescence was measured in a microplate luminometer. The value of luciferase activity was normalized to transfection efficiency monitored by the co-transfected β-galactosidase expression vector.

### Immunohistochemistry staining

Five-micro thick paraffin wax mouse-tissue sections were dewaxing by xylene and rehydrated by different concentration of ethanol. These mouse-tissue sections were incubated for overnight with IL-6 antibody (50 dilution, Santa Cruz, CA), and were washed for removal of unbound primary antibody. These sections were then stained with a polymer HRP conjugate secondary antibody rabbit antibody for 30 mins followed by reaction with diaminobenzidine for two minutes.

### Overexpression of microRNA-7 (miR-7) expression

TNBC cells were transfected with 1 μg of mimic-control and mimic-miR-7 plasmid vector [15]. TNBC cells were grown to 80% confluence in 12 well plates and transfected with *Trans*IT®-2020 on the following day. DNA and *Trans*IT®-2020 transfection reagent were pre-mixed with a ratio of 1:1 in serum-free medium for 20 mins and then applied to the cells. Serum was supplemented to the cells 6 hrs later. After 3-day transfection, the cells were subjected to indicate experiments.

### Chromatin immunoprecipitation (ChIP) assay

ChIP analysis was performed as described previously [31]. In brief, cells were treated, fixed and sheared. The DNA immunoprecipitated by c-Jun antibody was purified and then extracted with phenol-chloroform. The purified DNA pellet was subjected to PCR. PCR products were resolved by 2% agarose gel electrophoresis and visualized by UV. The primer sequences used for detecting IL-6 promoter region (−312 to −39) were as follows: Forward: 5′-GAACTGACCTGACTTACATA-3′ and reverse: 5′-TTGAGACTCATGGGAAAATCC-3′.

### RNA immunoprecipitation (RNAIP) assay

MDA-MB-231 or 231/Lap cells were transfected with 100 nM miR-7 mimic or inhibitor for 3 days, and then were fixed and sheared. The lystate was incubated with rabbit-IgG and Ago2 (cell signaling, Danvers, MA) antibody followed by immunoprecipitation with protein A/G beads at 4°C overnight. Then supernatant was removed, and beads were resuspended with RNAIP buffer followed by RNA isolation with TRIzol reagent. The purified RNA was subjected into reverse transcription with oligo (dT) primer. The immunoprecipitated Raf-1 mRNA level was examined in real-time PCR reaction with specific primers targeting at Raf-1 3′UTR as following: 5′- ACTAGTTTGACTTTGCACCTGTCTTC-3′ and 5′- AAGCTTACACCTAAATTTAATTTATT-3′.

### Statistic analysis

Data were displayed as means ±SEM. The significance of difference between the experimental and control groups was assessed by Student's *t* test. The difference is significant if the *p* value is < 0.05.
